# Innovation, implementation, and insight: new contributions to Dutch cardiovascular care

**DOI:** 10.1007/s12471-025-01990-z

**Published:** 2025-09-03

**Authors:** Pim van der Harst

**Affiliations:** Department of Cardiology, Division of Heart and Lungs, University Medical Centre Utrecht, Utrecht, The Netherlands

This issue of the *Netherlands Heart Journal* features 5 contributions across AI, registry science, congenital heart disease, health technology assessment, and critical care. Each article addresses a gap in clinical knowledge and exemplifies the depth and breadth of cardiovascular research in the Netherlands.

Van der Boon and Karper present a perspective on AI-assisted echocardiography, describing their implementation of the Us2.AI platform in two clinical settings. Their experience demonstrates how automated image analysis enhances quality and scalability in cardiac ultrasound while maintaining clinician oversight—a timely development amid rising imaging demands and AI’s expanding role [1].

Peijster et al. report the launch of the ENDOCOR registry for infective endocarditis. In a field shaped by limited evidence and heterogeneous practice, their work shows how national coordination improves data quality and care delivery. The pilot phase (150 patients, three centres) signals feasibility and relevance. As Derks et al. noted earlier, national registries support patient-centred care [2].

Immens et al. investigate anatomical features in patients undergoing percutaneous PFO closure after cryptogenic stroke. Although 80% had risk-enhancing features, one in five was accepted without them, underscoring the need for thorough interdisciplinary evaluation by dedicated Heart-Stroke Teams.

Saing et al. present an  assessment of the Dutch CardioVascular Alliance (DCVA), evaluating the potential impact of DCVA-supported consortia. Though exploratory, their study highlights the need to assess how collaboration advances national goals, such as reducing the cardiovascular burden by 25% by 2030.

Koolwijk et al. provide a systematic review and meta-analysis of anticoagulation in critically ill patients with trigger-induced atrial fibrillation. The study reveals wide variation in practice and no clear benefit or harm, highlighting significant clinical uncertainty and an unmet need.

Together, these articles exemplify integration of innovation and implementation in cardiovascular care. We thank the authors for their contributions and invite readers to explore these articles as a reflection of progress in Dutch cardiovascular medicine.
